# Shikonin inhibits cancer cell cycling by targeting Cdc25s

**DOI:** 10.1186/s12885-018-5220-x

**Published:** 2019-01-07

**Authors:** Shoude Zhang, Qiang Gao, Wei Li, Luwei Zhu, Qianhan Shang, Shuo Feng, Junmei Jia, Qiangqiang Jia, Shuo Shen, Zhanhai Su

**Affiliations:** 1 State Key Laboratory of Plateau Ecology and Agriculture, Qinghai University, 251# Ningda Road, Xining, 810016 Qinghai China; 2 Department of Pharmacy, Medical College of Qinghai University, 16# Kunlun Road, Xining, 810016 Qinghai China; 3 Qinghai Academy of Agriculture and Forestry Science, 251# Ningda Road, Xining, 810016 China

**Keywords:** Shikonin, Cdc25s, Anticancer, Cell cycle progression

## Abstract

**Background:**

Shikonin, a natural naphthoquinone, is abundant in Chinese herb medicine Zicao (purple gromwell) and has a wide range of biological activities, especially for cancer. Shikonin and its analogues have been reported to induce cell-cycle arrest, but target information is still unclear. We hypothesized that shikonin, with a structure similar to that of quinone-type compounds, which are inhibitors of cell division cycle 25 (Cdc25) phosphatases, will have similar effects on Cdc25s. To test this hypothesis, the effects of shikonin on Cdc25s and cell-cycle progression were determined in this paper.

**Methods:**

The in vitro effects of shikonin and its analogues on Cdc25s were detected by fluorometric assay kit. The binding mode between shikonin and Cdc25B was modelled by molecular docking. The dephosphorylating level of cyclin-dependent kinase 1 (CDK1), a natural substrate of Cdc25B, was tested by Western blotting. The effect of shikonin on cell cycle progression was investigated by flow cytometry analysis. We also tested the anti-proliferation activity of shikonin on cancer cell lines by MTT assay. Moreover, in vivo anti-proliferation activity was tested in a mouse xenograft tumour model.

**Results:**

Shikonin and its analogues inhibited recombinant human Cdc25 A, B, and C phosphatase with IC_50_ values ranging from 2.14 ± 0.21 to 13.45 ± 1.45 μM irreversibly. The molecular modelling results showed that shikonin bound to the inhibitor binding pocket of Cdc25B with a favourable binding mode through hydrophobic interactions and hydrogen bonds. In addition, an accumulation of the tyrosine 15-phosphorylated form of CDK1 was induced by shikonin in a concentration-dependent manner in vitro and in vivo. We also confirmed that shikonin showed an anti-proliferation effect on three cancer cell lines with IC_50_ values ranging from 6.15 ± 0.46 to 9.56 ± 1.03 μM. Furthermore, shikonin showed a promising anti-proliferation effect on a K562 mouse xenograph tumour model.

**Conclusion:**

In this study, we provide evidence for how shikonin induces cell cycle arrest and functions as a Cdc25s inhibitor. It shows an anti-proliferation effect both in vitro and in vivo by mediating Cdc25s.

## Background

**Fig. 1:**
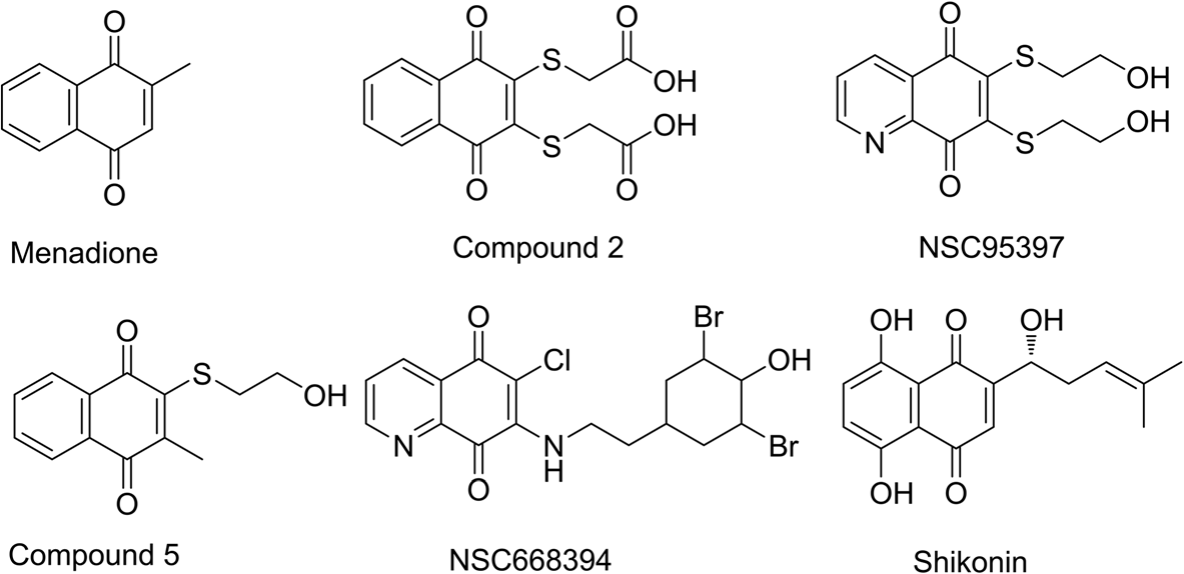
Structures of quinone-type inhibitors of Cdc25s and shikonin

Dual-specificity protein phosphatases (DSP) Cdc25s (Cdc25A, Cdc25B, and Cdc25C) have an essential role in cell cycle progression via controlling the phosphorylation state of their natural substrates cyclin-dependent kinases (CDKs). Overexpression of Cdc25s and over-activation of CDKs are involved in tumour-associated cell-cycle aberration [1]. Therefore, Cdc25s is currently considered to be a promising anticancer target [2–4]. Several inhibitors of Cdc25s with antitumour activity have been reported [5, 6]. Most potent small molecule inhibitors of the Cdc25 phosphatases are quinone-derived compounds, such as menadione (vitamin K_3_) [7], compound 2 [8], NSC95397 [9], compound 5 [10], and NSC668394 [11] (Fig. 1). This type of compound was hypothesized to inhibit the activity of Cdc25s by oxidation of the catalytically essential cysteine residue in the enzyme’s active site through production of reactive oxygen species [12].

The naphthoquinone-type natural product shikonin was derived from the root of *Lithospermum erythrorhizon,* which has broad applications in Traditional Chinese Medicine [13, 14]. Over the past few decades, a number of studies have demonstrated multiple biological effects of shikonin. It has been reported to have anti-HIV [14], anti-inflammatory [15, 16], antibacterial, and anticancer [17–20] activities. Among these activities, the anticancer activity, especially the induction of apoptosis and necroptosis, is well reported [13, 19–23]. However, the key target is still unclear. Shikonin has a similar chemical skeleton to that of the quinone-type inhibitors of Cdc25s. Therefore, we hypothesized that shikonin will have similar effects on Cdc25s. To test this hypothesis, the effects of shikonin on Cdc25s and related biofunction were confirmed in this paper.

## Methods

### Chemicals

Shikonin and its analogues are natural products that were purchased from Herbest, Inc. (Baoji, Shanxi, China). All other chemicals were purchased from Sigma-Aldrich (Shanghai, China) unless otherwise noted.

### Measurement of phosphatase inhibitory activity of shikonin and its analogues

CycLex® protein phosphatase Cdc25A, -B and -C fluorometric assay Kit (CycLex, Cat. No. CY-1352, CY-1353, CY-1354) was used to test the enzyme inhibition rate of shikonin and its analogues for Cdc25A, -B and -C. In summary, dual-specificity phosphatase activity was measured in a 96-well microtiter plate using O-methylfluorescein phosphate (OMFP) as a substrate. 5 μL (0.1 μg/μL) of purified recombinant Cdc25s (Cdc25A, -B and -C) was mixed with 40 μL of assay mixture and incubated with 5 μL of the test compound at various concentrations in a well. Then, 25 μL of stop solution was added. Fluorescence was measured at an excitation wavelength of 485 nm and an emission wavelength of 530 nm using a fluorescence microplate reader (BioTek Instruments, Inc., Winooski, VT, USA). Menadione is a quinone-type inhibitor of Cdc25s [7] that was used as a positive control here.

For the dialysis assay, the enzyme-inhibitor complex including 0.2 μM Cdc25B and 50 μM shikonin was dialyzed against 5000-fold of the assay buffer for the indicated period of time. At the end of each dialysis, Cdc25B activity was determined as described above.

### Molecular modelling

The docking method used is described in a previous work [24]. In summary, molecular modelling was performed using Maestro 9.0. The X-ray structure of Cdc25B (PDB ID: 1QB0) was downloaded from the Protein Data Bank (PDB, https://www.rcsb.org) and prepared with the “Protein Preparation Wizard” workflow with default settings. The grid-enclosing box was generated within 10 Å of Cys473 in the refined crystal structure. The ligand structure was prepared with the Ligprep module. Finally, shikonin was docked into Cdc25B using Glide (version 5.5) in extra precision (XP) mode with default settings [25]. Favourable binding poses were selected according to the docking score and view check.

### Cell lines and culture conditions

K562 cells (myelogenous leukaemia cell line), MCF-7 cells (breast cancer cell line) and HeLa cells (cervical cancer cells) were obtained from the Chinese Academy of Sciences Cell Bank (Shanghai, China). The catalogue numbers of these cell lines are TCHu191, TCHu74 and TCHu187, respectively. The temperature-sensitive FT210 cell line (tsFT210) is a mouse breast cancer cell line that is widely used for studying cell cycle progression. Intracellular CDK1 protein of tsFT210 is inactive at 39 °C because of two point mutations on the cdc2 gene, which leads to it being easily controlled at different cell cycle phases through changes in temperature [26]. This cell line was kindly provided by Dr. Rongcai Yue from his laboratory (School of Pharmacy, Second Military Medical University). All cell lines were kept in the logarithmic growth phase in 5% CO_2_ at 37 °C with RPMI-1640 medium supplemented with 10% FBS and 1% penicillin G-streptomycin in a humidified chamber at 5% CO_2_.

### Western blotting

The phosphorylation status of CDK1 was analysed by Western blotting as described in our previous work [24]. In summary, tsFT210 cells (1 × 10^6^) were treated with shikonin (0, 1, 5 and 25 μM) for 4 h followed by collection and suspension in lysis buffer (150 mM NaCl, 50 mM Tris, 0.02% NaN_3_, 0.01% phenylmethylsulfonyl fluoride, 0.2% aprotinin, and 1% TritonX-100, pH 8.0) containing a protease inhibitor cocktail (Thermo Scientific) for 30 min at 4 °C. The protein concentration in cell lysates was confirmed by the Bradford method. Fifty micrograms of protein per lane was resolved on 10% SDS polyacrylamide gels, and the bands were transferred to PVDF membranes (Millipore). Nonspecific reactivity was blocked by 5% non-fat milk prepared in TBST (10 mM Tris, 150 mM NaCl, 0.05% Tween-20, pH 7.5) at room temperature for 1 h. The protein signals were captured with primary antibodies and the secondary antibodies according to the manufacturers’ instructions. In this process, the protein β-actin was used to normalize target protein. All antibodies used in this paper were purchased from Cell Signaling Technology (Inc, China).

### Intracellular reactive oxygen species assay

Reactive oxygen species (ROS) were detected with the fluorescent probe 2′,7′-dichlorofluorescin diacetate (DCFH-DA) as described by others [18, 27]. Briefly, MCF-7 cells or tsFT210 (synchronized at G2 phase) cells were plated in 96-well plates and loaded with 20 μM DCFH-DA for 30 min at 37 °C in culture medium. Cells were washed with PBS three times after incubation and then treated with shikonin at concentrations of 5 μM and 25 μM for 4 h. Untreated cells were used as control. Fluorescence was measured using a fluorescence microplate reader (BioTek Instruments, excitation wavelength: 485 nm, emission wavelength: 528 nm). The ROS levels were expressed as RFU (relative fluorescence unit).

### Cell cycle analysis

Cell cycle analysis was performed as described previously [28]. Briefly, tsFT210 cells were plated at 1 × 10^5^ cells/well (24-well plate) and maintained at 32 °C. Cell proliferation was blocked at the G2 phase by increasing the temperature from 32 to 39 °C and treating for 17 h. Then, the cells were released at 32 °C and immediately treated with shikonin. The harvested cells were stained with staining buffer (50 μg/ml propidium iodide, 0.1% sodium citrate and 0.2% NP-40) and analysed by Flow Cytometry (BD Biosciences). A final concentration of 0.5% DMSO was used for all compounds and as a negative control. Nocodazole, a potent mitotic blocker that arrests cells at the G_2_/M phase, was used as a positive control at a concentration of 100 nM.

### Anti-proliferation assay

The anti-proliferation activity of shikonin against cancer cells was tested by the MTT method in 96-well plates. First, 4 × 10^3^ cells were seeded per well and treated with the shikonin with serial concentrations for 48 h. Then, the cells were incubated with 10 μL of MTT solution (5 mg/mL in PBS) for an additional 2–4 h at 37 °C. The formazan crystals were dissolved with 100 μL of DMSO after removing the supernatant. The absorbance at 570 nm was measured by a BioTek Synergy 2 plate reader (BioTek Instruments, Inc., Winooski, VT, USA). Doxorubicin, a chemotherapy medication used to treat cancer and having a broad spectrum of anticancer activities [29], was used as a positive control here.

### Xenograph tumour model in mice

The mouse model was constructed according to our previous work [24]. Balb/c mice (female, 5 weeks old), purchased from Shanghai SLAC Laboratory Animal Co., LTD (Shanghai, China), were subcutaneously injected into a 100 μL K562 cell suspension (5 × 10^6^/mL, PBS) in the right flank of mice. We started treating the mice with shikonin at 1, 5, or 10 mg/kg or vehicle containing 5% DMSO (control group) by intraperitoneal injection every other day when the tumour’s volume reached approximately 200 mm^3^. The long and short dimensions of tumour size were measured by a digital calliper at days 1, 4, 8, 11, 16, 21, and 24 after treatment. The volume was calculated with the formula (long dimension) × (short dimension)^2^/2. The mice were euthanized 2 days after the last treatment by the method of cervical dislocation.

### Statistical analysis

Statistical analyses were performed using PRISM software. All quantitative values are given as the means ± SEM. An unpaired Student’s t test was used to evaluate the difference between two different treatments, and *p* < 0.05 was considered statistically significant. The statistical significance of differences in the survival of mice from the different groups was determined by the log-rank test using the same program.

## Results

### Shikonin induce irreversible inhibition of human recombinant CDC25 phosphatases

Using the protein phosphatase Cdc25 combo fluorometric assay kit, shikonin and its analogues in vitro inhibited recombinant human Cdc25A, -B, and -C phosphatases in a concentration-dependent manner with IC_50_ values ranging from 2.14 to 14.32 μM (Table 1). The positive control menadione led to an inhibition of 4.12 ± 0.87, 5.37 ± 0.45, and 5.13 ± 0.24 μM, respectively.

**Table 1:** IC_50_ values of shikonin and analogues for inhibition of recombinant human protein phosphatases

Structure	R	Name	Cdc25A (μM)	Cdc25B (μM)	Cdc25C (μM)
	-OH	Shikonin	2.14 ± 0.21	5.82 ± 0.37	4.78 ± 0.18
	-H	Deoxyshikonin	3.22 ± 0.76	7.32 ± 0.45	6.33 ± 0.65
	-OCOCH_3_	Acetylshikonin	3.92 ± 0.66	3.87 ± 0.68	4.67 ± 0.34
	-OCOCH(CH_3_)_2_	Isobutylshikonin	6.79 ± 1.02	7. 86 ± 0.23	5.89 ± 0.43
	-OCOCH_2_CH(CH_3_)_2_	Isovalerylshikonin	9.53 ± 0.78	11. 23 ± 1.22	10.98 ± 0.97
	-OCOCH=CH(CH_3_)_2_	β,β-Dimethylacrylshikonin	4.67 ± 0.85	8.56 ± 0.67	10. 58 ± 1.13
	**-**OCOCH(CH_3_)CH_2_CH_3_	α-Methyl-η-butylshikonin	11.24 ± 1.45	14.32 ± 1.27	13.45 ± 1.45

All values are micromolar concentrations and are the mean ± S.E.M. of three or more independent determinations

**Fig. 2:**
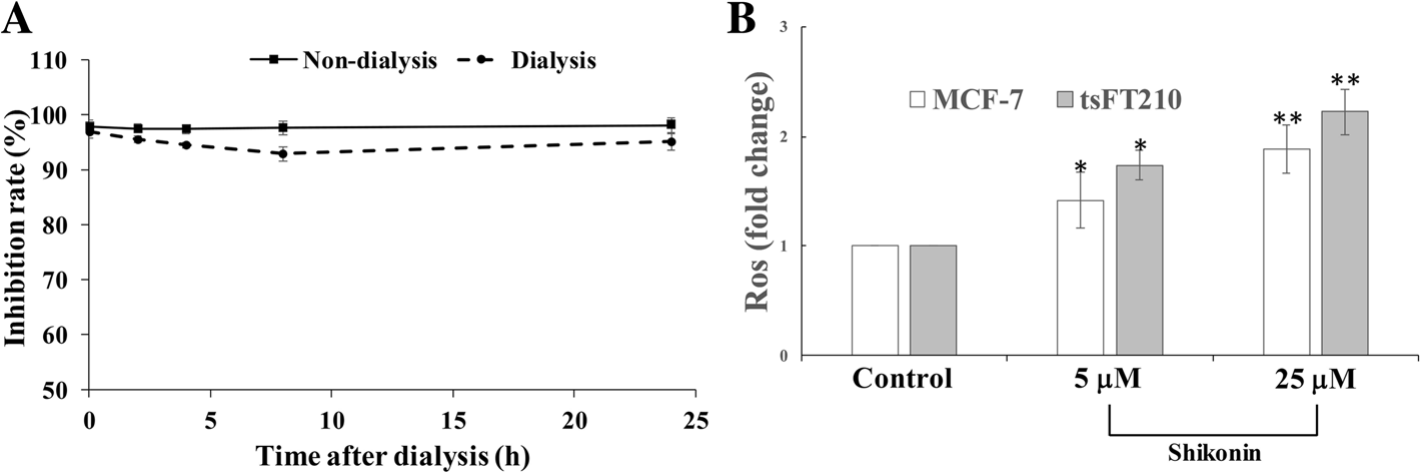
Shikonin inhibits Cdc25B irreversibly in vitro and induces ROS production in MCF-7 cells. **a** Activity of shikonin-treated Cdc25B after dialysis. **b** ROS production in MCF-7 and tsFT210 cells after treatment with shikonin. Data points represent fold change of three independent experiments. *P < 0.05; **P < 0.01 compared with the control group

Previous research revealed that the quinone-type inhibitors of Cdc25 phosphatases play a role through irreversible oxidation of the catalytic cysteine of Cdc25B [30, 31]. To identify the inhibition pattern of shikonin, dialysis was used to study the reversibility of shikonin action. As shown in Fig. 2a, after dialysis for 2, 4, 6, and even 24 h, the activity of Cdc25B was not rescued from inhibition by shikonin, indicating that the Cdc25B was inhibited irreversibly. In addition, to confirm whether the shikonin has similar mechanism with other quinone-type inhibitors, the ROS content was measured in cells treated with or without shikonin. After treating with shikonin, intracellular ROS in MCF-7 and tsFT210 cells increased significantly compared with those of control cells (Fig. 2b), suggesting that Cdc25s inhibition by shikonin could be caused by redox cycling of the shikonin similar to that of other quinones.

### Molecular model of shikonin interactions with the Cdc25B catalytic domain

**Fig. 3:**
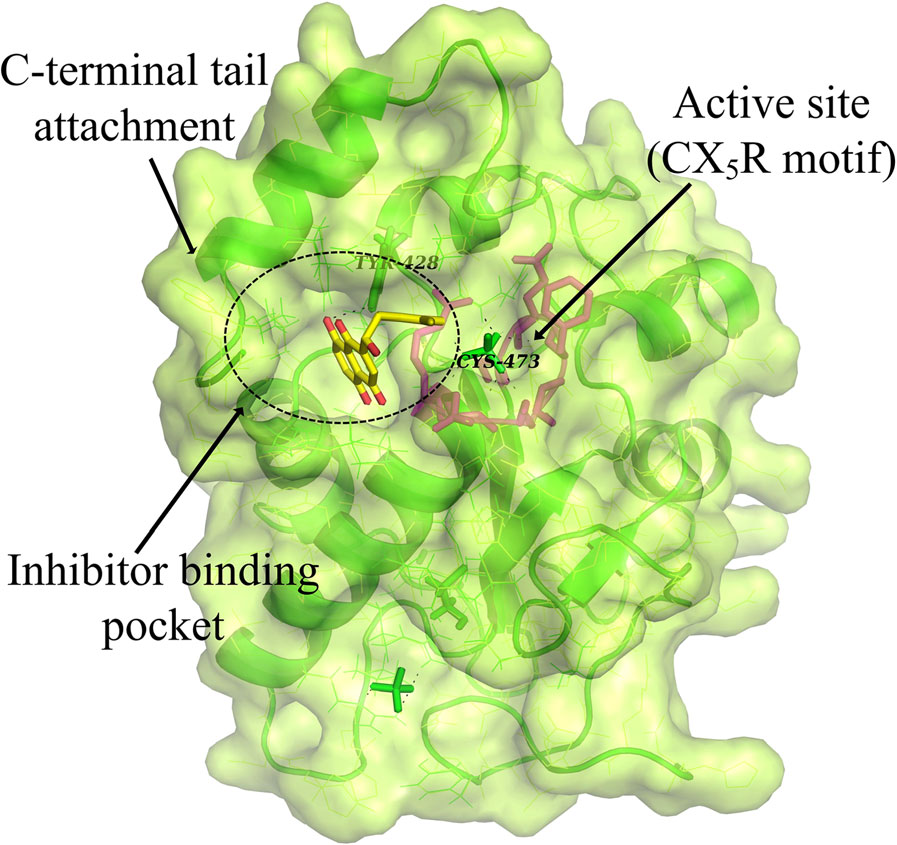
Binding mode of shikonin in the Cdc25B binding cavity. Shikonin (yellow) and key interacting residues (green) are represented as stick models with the protein as a surface. H-bonds are shown as dashed black lines. The figure was generated using Pymol from Protein Data Bank ID 1QB0

In order to evaluate the binding mode and affinity of shikonin with Cdc25B, molecular modelling was performed using the docking program Glide. X-ray crystallography showed that the catalytic domain of Cdc25B contains the canonical HCX_5_R PTPase catalytic-site motif [32]. The phosphorylated amino acids of the substrate usually bind to this loop through forming hydrogen bonds to arginine R. The quinone-type inhibitor was supposed to bind the inhibitor binding pocket, which was beside the active site (Fig. 3), and induce oxidation of the catalytic cysteine through redox cycling reaction [1]. After docking the shikonin into the above binding loop around cys473, we found that the shikonin bound to the inhibitor binding pocket similar to other quinone-type inhibitors. Figure 2 shows the most energetically favourable binding pose from docking results. In such a binding mode, the benzene ring of shikonin is involved in hydrophobic interactions with the pocket residues. In addition, a hydrogen bond between one oxygen of the naphthoquinone core and Tyr428 was predicted.

### Shikonin inhibits cell cycle progression

**Fig. 4:**
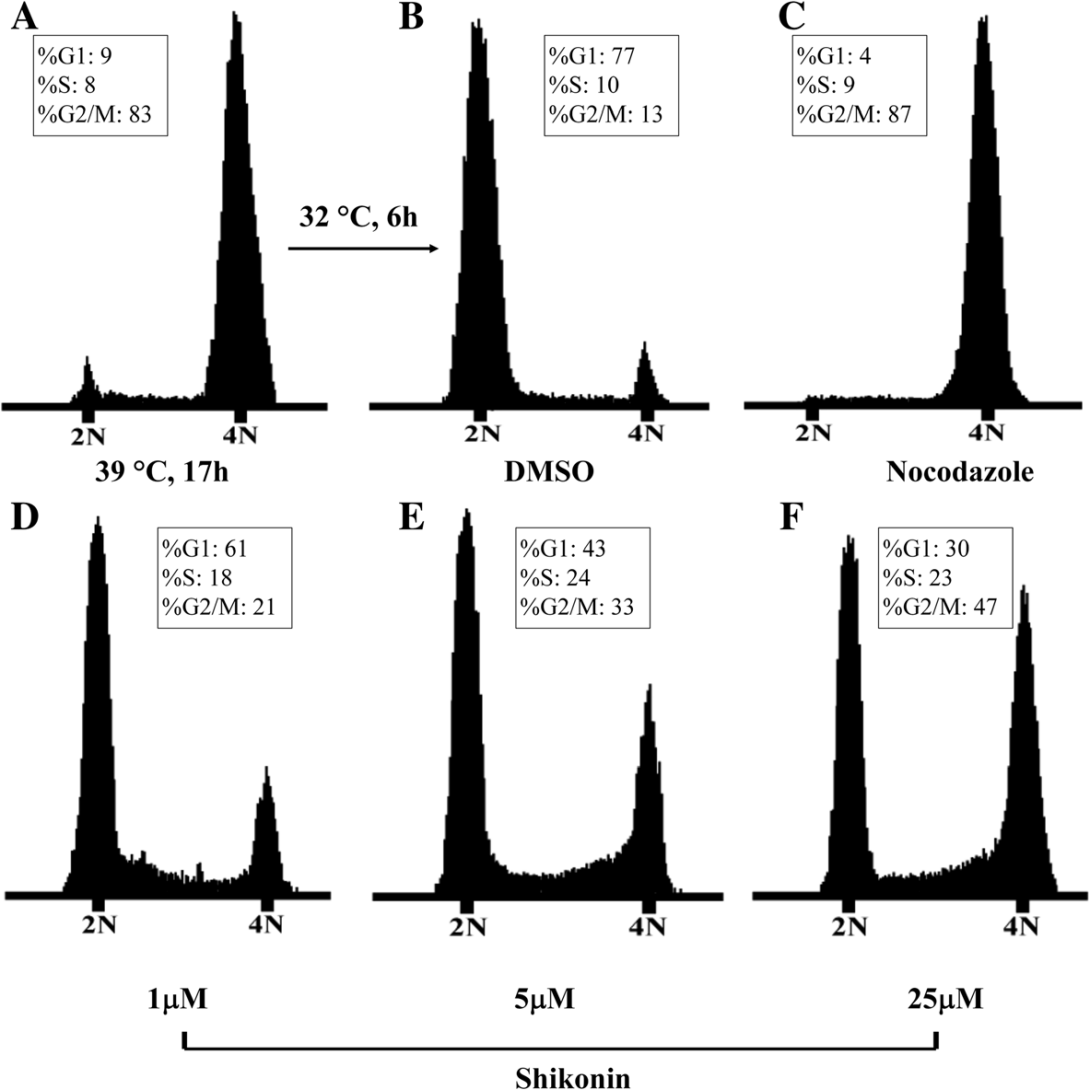
Cell cycle analysis of tsFT210 cells in the absence or presence of shikonin. **a** G2/M-arrested cells after a temperature shift for 17 h at 39 °C. **b** DMSO-treated cells after a temperature shift for 4 h at 32 °C. **c** Cells treated with 100 nM nocodazole. **d-f** Cells treated with 1–25 μM shikonin. Data are representative of two independent experiments

Inhibition of Cdc25s results in hyper-phosphorylation of CDKs and cell cycle arrest. Therefore, the impact of shikonin on cell cycle progress was investigated. The tsFT210 cell line has been widely used for studying cell cycle progression because it can be easily controlled at different cell cycle phases through changing temperature [26]. The synchronized tsFT210 cells were then treated with the indicated concentration of shikonin and nocodazole (a potent mitotic blocker) or 1% DMSO for 6 h. The positive control nocodazole arrested the cells at the G_2_/M phase. Comparatively, the cells treated with shikonin were blocked at the G_2_/M phase in a concentration-dependent manner, whereas the DMSO-treated control cells had completed mitosis and had entered the following cell cycle (Fig. 4). Such results indicate that shikonin can target and delay cell cycle progression at the G2/M phase.

### Shikonin inhibits CDK1 dephosphorylation

**Fig. 5:**
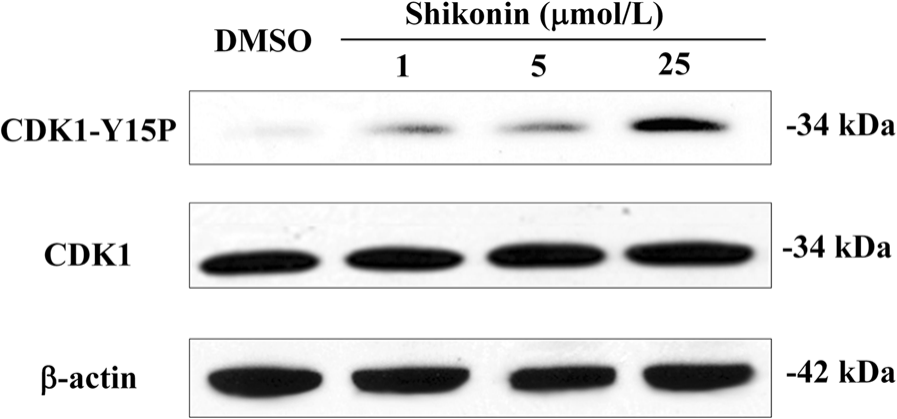
Inhibition of CDK1 dephosphorylation caused by shikonin. Cells in G2/M phase were treated with the indicated concentrations of shikonin and DMSO for 4 h and then harvested. Samples were processed for Western blot analysis. Data are representative of two independent experiments

Endogenous Cdc25s controls the cell cycle through dephosphorylation of their natural substrate CDKs [2]. Therefore, CDK1 protein will be hyper-phosphorylated if the CDC25s are inhibited. In order to confirm whether shikonin inhibits the activity of intracellular Cdc25 phosphatases, the phosphorylation status of CDK1 was analysed by Western blotting. As shown in Fig. 5, shikonin at 1 μM, 5 μΜ, and 25 μM induced an accumulation of the tyrosine 15-phosphorylated form of CDK1. These results suggested that shikonin downregulated the activity of Cdc25s, leading to hyper-phosphorylation of CDK1 in cultured cells.

### Shikonin inhibits in vitro tumour cell proliferation

To evaluate the anti-proliferation activity of shikonin for different cancer cells, four cancer cell lines (MCF-7, HeLa, K562, and tsFT210) were selected to investigate the anti-proliferation activity. The viability of cells was evaluated by the MTT method. As shown in Table 2, shikonin markedly inhibits the proliferation of MCF-7 cells (IC_50_: 6.82 ± 0.76 μM), HeLa cells (IC_50_: 9.56 ± 1.03 μM), K562 cells (IC_50_: 6.15 ± 0.46 μM) and tsFT210 cells (8.97 ± 0.87 μM) compared with the negative control. Doxorubicin was used as a positive control and showed normal anticancer activity according to a previous report.

**Table 2:** Inhibitory activity of shikonin on tumour cells via MTT assay (IC_50_, μM, *n* = 3, mean ± SD)

Compound	Cell Line			
	MCF-7	HeLa	K562	tsFT210
Shikonin	6.82 ± 0.76	9.56 ± 1.03	6.15 ± 0.46	8.97 ± 0.87
Doxorubicin	0.28 ± 0.03	0.43 ± 0.06	0.51 ± 0.12	0.56 ± 0.14

### Shikonin has anti-tumour effects in vivo by inhibiting CDK1 dephosphorylation

**Fig. 6:**
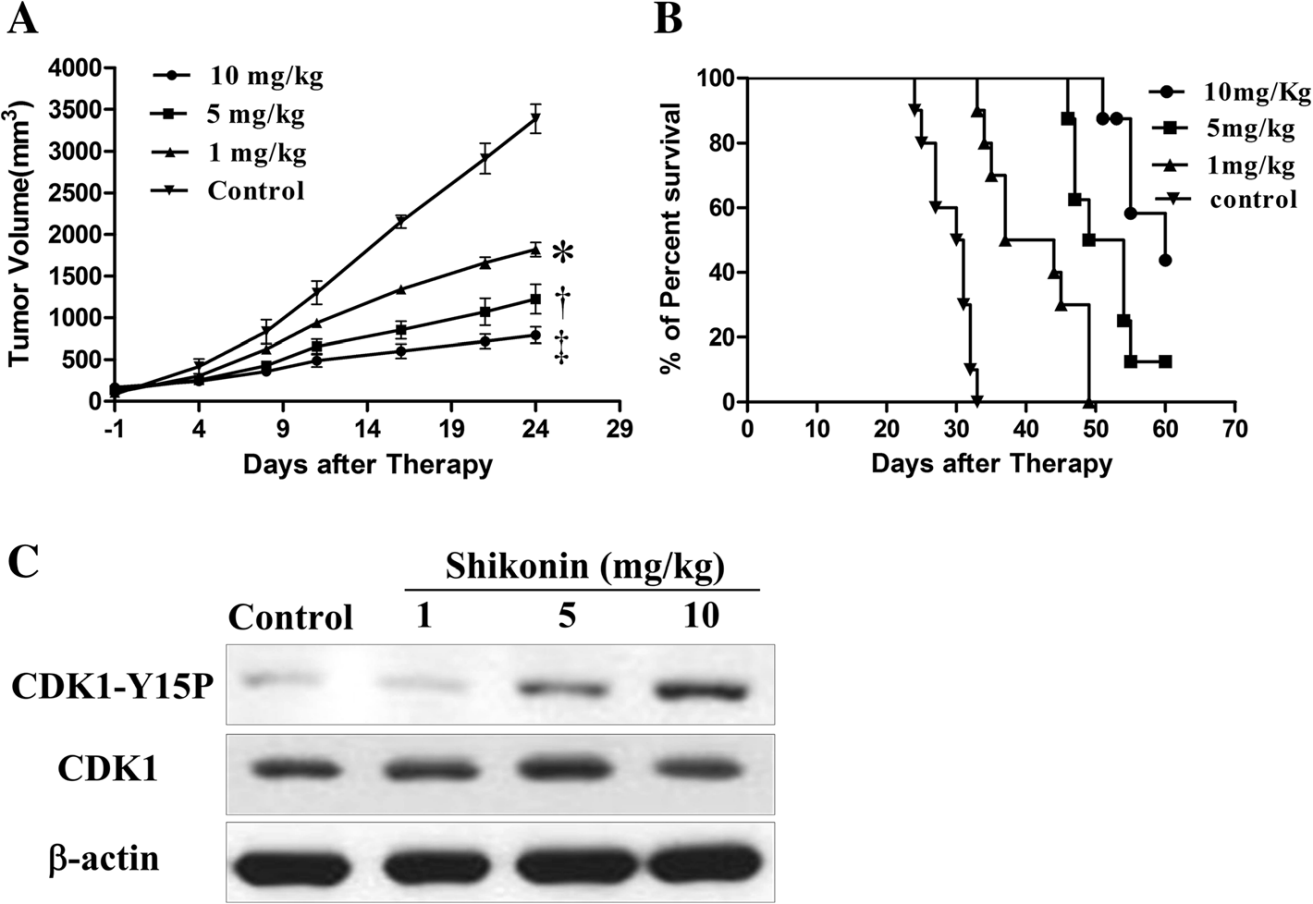
Shikonin inhibits tumour growth in vivo in the K562-bearing mice by affecting the phosphorylation of CDK1 (*n* = 10/group). **a** Tumour volume plot of K562-bearing mice treated with vehicle or shikonin at 1, 5, or 10 mg/kg by oral gavage for 21 days. The tumours were measured twice per week. The data are represented as the mean ± SEM. Tumour growth was inhibited significantly after treatment with shikonin compared with the control group. *P < 0.05; †*P* < 0.01; ‡*P* < 0.001 compared with the control group. **b** Kaplan-Meier survival plot of the K562-bearing nude mice. Survival of the K562-bearing nude mice was prolonged in the shikonin-treated groups compared with control group. **c** The phosphorylation level of CDK1 is affected by shikonin.

Xenograph tumour model mice were used to evaluate if shikonin can inhibit tumour growth in vivo. K562-bearing mice were treated with shikonin at different doses and vehicle for 21 days. The tumour sizes were recorded in this process. As shown in Fig. 5a, shikonin concentration-dependently inhibited tumour growth. Higher doses of shikonin had a better inhibitory effect and longer observed survival time (Fig. 6a, 6b). In the process of this experiment, the tumour growth of mice from higher dose groups was significantly inhibited compared with those of the control and low-dose groups. These results suggest that shikonin has profound anticancer activity in vivo. Moreover, the protein levels of phosphorylated CDK1 in primary tumour tissues obtained from the mice were significantly increased after treatment by shikonin compared with that of the control group, especially for the 5 and 10 mg/kg groups (Fig. 6c). This means that the shikonin still participates in the cell cycle process in vivo.

## Discussion

Overexpression of CDC25s, which leads to an irregular cell cycle, is often present in cancer cells. They control the cell cycle by activating CDKs. Therefore, CDC25s are considered attractive targets for anticancer drug discovery. Several potent CDC25s inhibitors have been identified; however, few of them have entered clinical trials. Shikonin, a natural naphthoquinone, has a similar structure to that of the well-reported quinone-type inhibitors of CDC25s. Hence, we hypothesized that shikonin will have similar effects on Cdc25s. To test this hypothesis, the effects of shikonin on Cdc25s and related biofunction were proved in this work.

Shikonin and six analogues showed considerable inhibition for CDC25s using OMFP as substrate. The main difference between these analogues and shikonin is the R groups (Table 1), but they have similar inhibition for CDC25s. Therefore, we suggest that the R groups have little effect on the activity and that the pharmacological group is the skeleton of naphthoquinone. Finally, shikonin was selected for further research because it showed the best inhibition for CDC25s compared with other analogues and can be generated from other analogues by hydrolysing the R group. Shikonin binds to the same pocket of Cdc25B as other quinone-type inhibitors according to the molecular modelling results. Together with the unaltered activity of Cdc25B after dialysis and increasing ROS content after treatment with shikonin, we suggested that shikonin inhibited Cdc25B similar to other quinone-type inhibitors, which were hypothesized to disrupt Cdc25s phosphatase activity by oxidation of the catalytically essential cysteine residue in the enzyme’s active site through production of reactive oxygen species [23].

Endogenous Cdc25s control the cell cycle through dephosphorylating their natural substrate CDKs. Therefore, the impacts of shikonin on cell cycle progress and phosphorylation state of CDK1 were investigated. We proved that shikonin could block the cell cycle of tsFT210 at the G2/M phase and effectively inhibit dephosphorylation of CDK1 in vitro.

We also evaluated the anti-proliferation activity of shikonin both in vitro and in vivo. Shikonin showed broad spectrum anticancer activity after evaluating the anti-proliferation activity for four cancer cell lines. Moreover, tumour growth of xenograph tumour model mice was significantly inhibited by shikonin. To confirm the in vivo activity of shikonin was through inhibition of the Cdc25s, we also checked the phosphorylation state of CDK1 in tumour tissue from xenograph tumour model mice, and the results showed that shikonin could inhibit dephosphorylation of CDK1 in tumour tissue also, which suggested that shikonin could also participate in the Cdc25s-related cell cycle pathway in vivo.

Shikonin reportedly exerts anticancer effects with less drug resistance [33], which can be attribute to its multiple targets. ROS generation, inactivation of NF-κB, upregulation of p53 and p21, and activation of caspases may be involved in the anticancer mechanisms of shikonin [34–38]. In brief, shikonin disrupts the cancer cell lines by inhibiting either the necroptosis or apoptosis pathway. Cancer cells died predominantly through the necroptotic pathway when they were co-treated with shikonin and the apoptosis inhibitor Z-VAD-FMK, while apoptosis became a selective route for cell death when the cancer cells were co-treated with shikonin and necroptotic inhibitor Nec-1 [39]. Moreover, shikonin and its analogues have been reported can induce cell cycle arrest, but the mechanism is still unclear [27]. Here, we identified shikonin as an inhibitor of Cdc25s that plays an essential role in cell cycle progression.

**Fig. 7:**
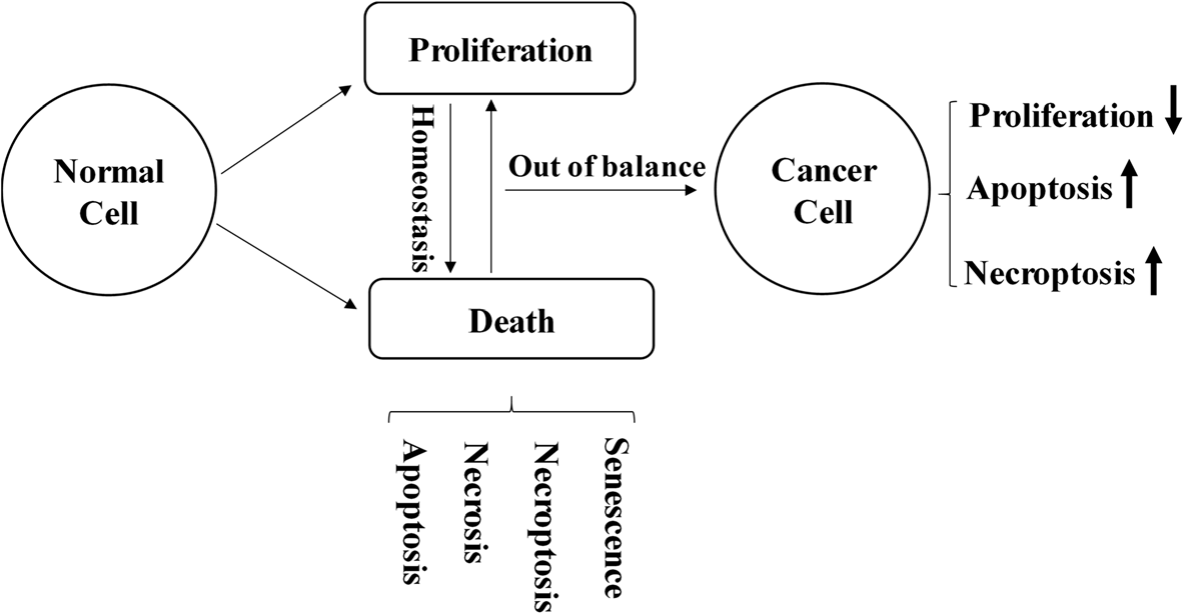
Schematic of the coordination between cell proliferation and death. ↓: inhibited by shikonin, ↑: promoted by shikonin

The normal development of tissue depends on the homeostasis and balance between cell proliferation and death (Fig. 7). When out of balance, as generated by inappropriate proliferation or inhibition of apoptosis and necroptosis pathways, cancer usually results [40]. If one pathway was inhibited or key proteins of it mutated, shikonin can also play an anticancer role through two other pathways, which could explain the lower drug resistance of shikonin.

## Conclusions

In conclusion, shikonin was first identified as an inhibitor of Cdc25s in the current study. It was shown to inhibit cancer cell growth by arresting the cell cycle through binding to Cdc25s in vitro and in vivo. Together with previous research, shikonin is shown to have anticancer function through regulation of the apoptosis, necroptosis, and cell cycle pathways.

## Abbreviations

CDK: Cyclin-dependent kinases
ROS: Reactive oxygen species
