# Correction: Shikonin inhibits cancer cell cycling by targeting Cdc25s

**DOI:** 10.1186/s12885-023-10976-2

**Published:** 2023-05-19

**Authors:** Shoude Zhang, Qiang Gao, Wei Li, Luwei Zhu, Qianhan Shang, Shuo Feng, Junmei Jia, Qiangqiang Jia, Shuo Shen, Zhanhai Su

**Affiliations:** 1 State Key Laboratory of Plateau Ecology and Agriculture, Qinghai University, 251# Ningda Road, Xining, 810016 Qinghai China; 2 Department of Pharmacy, Medical College of Qinghai University, 16# Kunlun Road, Xining, 810016 Qinghai China; 3 Qinghai Academy of Agriculture and Forestry Science, 251# Ningda Road, Xining, 810016 China

**Correction: BMC Cancer 19, 20 (2019)**

**https://doi.org/10.1186/s12885-018-5220-x**

Following publication of the original article [1], the authors found an error when checking the raw data. The anti-CDC25B activity of two compounds with Western Blot experiments in the same batch were tested, and there was an overlap between the two batches when processing the data. To make sure of the certainty of the conclusions, we conducted another experiment and found that the results were consistent with those in the article. The corrected Western Blot results for Figs. 5 and 6C are given in this correction article.

**Fig. 5:**
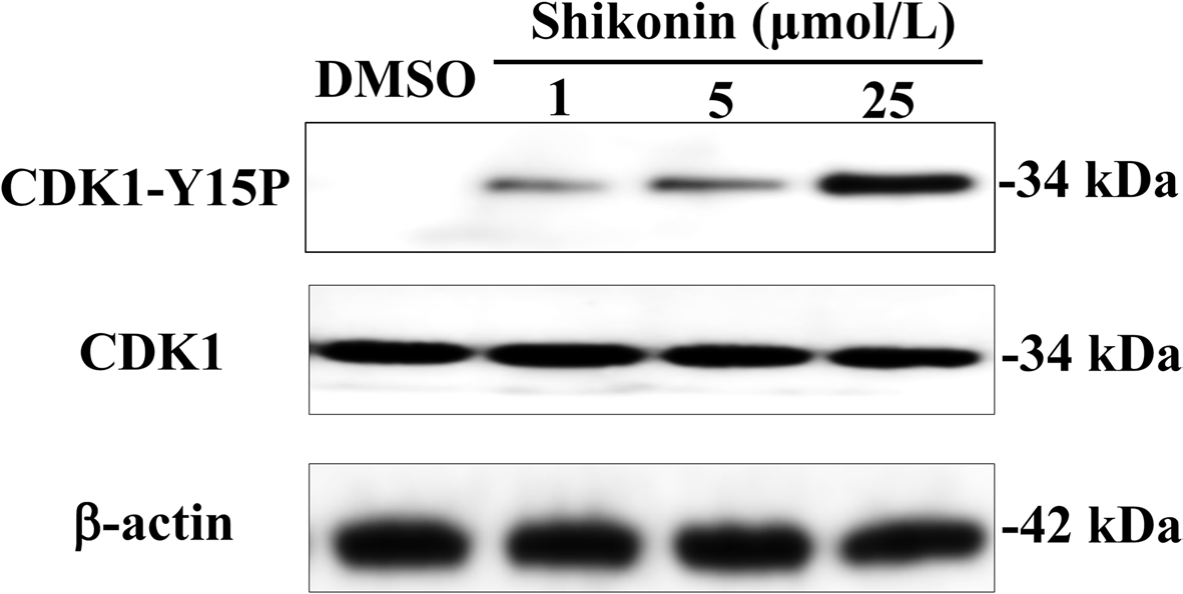
Inhibition of CDK1 dephosphorylation caused by shikonin. Cells in G2/M phase were treated with the indicated concentrations of shikonin and DMSO for 4 h and then harvested. Samples were processed for Western blot analysis. Data are representative of two independent experiments

**Fig. 6:**
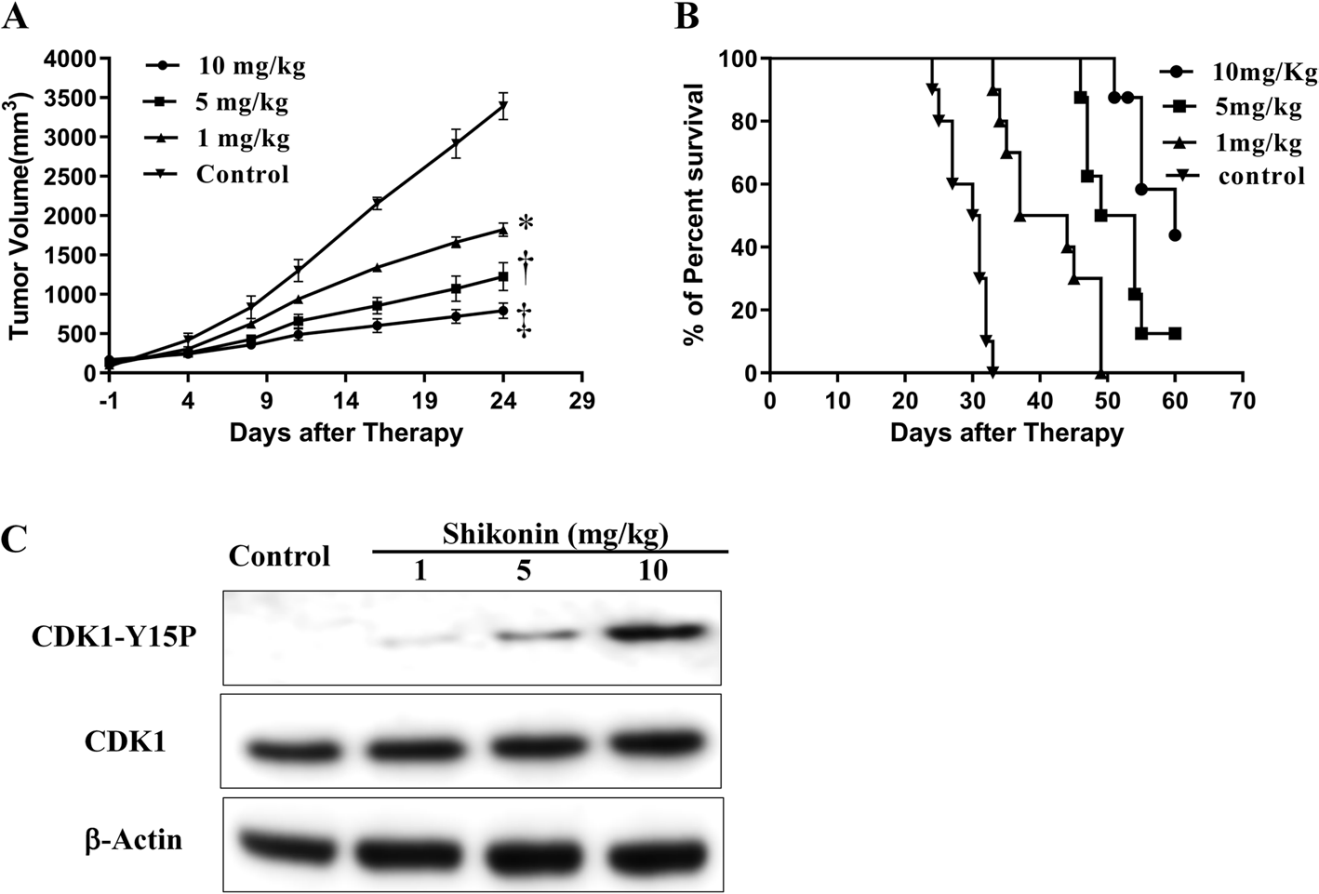
Shikonin inhibits tumour growth in vivo in the K562-bearing mice by affecting the phosphorylation of CDK1 (*n* = 10/group). **a** Tumour volume plot of K562-bearing mice treated with vehicle or shikonin at 1, 5, or 10 mg/kg by oral gavage for 21 days. The tumours were measured twice per week. The data are represented as the mean ± SEM. Tumour growth was inhibited significantly after treatment with shikonin compared with the control group. *P < 0.05; †*P* < 0.01; ‡*P* < 0.001 compared with the control group. **b** Kaplan–Meier survival plot of the K562-bearing nude mice. Survival of the K562-bearing nude mice was prolonged in the shikonin-treated groups compared with control group. **c** The phosphorylation level of CDK1 is affected by shikonin
